# Biomarkers Associated with Thrombosis in Patients with Peripherally Inserted Central Catheter: A Systematic Review and Meta-Analysis

**DOI:** 10.3390/jcm12134480

**Published:** 2023-07-04

**Authors:** Patrícia Cristina Cardoso, Eneida Rejane Rabelo-Silva, Patricia Martins Bock, Vineet Chopra, Marco Aurélio Lumertz Saffi

**Affiliations:** 1 Graduate Program in Cardiology and Cardiovascular Sciences, Universidade Federal do Rio Grande do Sul, Porto Alegre 90035-003, RS, Brazil; patriciacardoso@hcpa.edu.br (P.C.C.); eneidarabelo@gmail.com (E.R.R.-S.); 2 School of Nursing, Universidade Federal do Rio Grande do Sul (UFRGS), Porto Alegre 90620-110, RS, Brazil; 3 Cardiology Department, Hospital de Clínicas de Porto Alegre (HCPA), Porto Alegre 90035-903, RS, Brazil; 4 Nursing Department, Faculdades Integradas de Taquara (FACCAT), Taquara 95612-150, RS, Brazil; pmbock@terra.com.br; 5 Department of Medicine, University of Colorado, Denver, CO 80045, USA; vineet.chopra@cuanschutz.edu

**Keywords:** systematic review, catheterization, peripheral, thrombosis

## Abstract

Background: The measurement and identification of plasma biomarkers can support the estimation of risk and diagnosis of deep vein thrombosis (DVT) associated with the use of a peripherally inserted central catheter (PICC). Objectives: This systematic review and meta-analysis aimed to identify the association between the levels of potential biomarkers that reflect the activation of the blood system, long-term vascular complications, inflammatory system, and the occurrence of PICC-related DVT. Methods: Seven electronic databases (Embase, Web of Science, Medline, Scopus, Cinahl, Cochrane Central Register of Controlled Trials, and ERIC) were searched to identify literature published until December 2022. Studies were required to report: (I) adult and pediatric patients, outpatient or admitted to clinical, surgical, or ICU with PICC; (II) patients with PICC-related DVT and patients without PICC-related DVT as a comparator; and (III) at least one biomarker available. The Newcastle–Ottawa Scale was used to evaluate the quality of the studies. Study precision was evaluated by using a funnel plot for platelets level. We provided a narrative synthesis and meta-analysis of the findings on the biomarkers’ outcomes of the studies. We pooled the results using random effects meta-analysis. The meta-analysis was conducted using Review Manager software v5.4. This systematic review is registered in PROSPERO (CRD42018108871). Results: Of the 3564 studies identified (after duplication removal), 28 were included. PICC-related DVT was associated with higher D-dimers (0.37 μg/mL, 95% CI 0.02, 0.72; *p* = 0.04, I^2^ = 92%; *p* for heterogeneity < 0.00001) and with higher platelets (8.76 × 10^9^/L, 95% CI 1.62, 15.91; *p* = 0.02, I^2^ = 41%; *p* for heterogeneity = 0.06). Conclusions: High levels of D-dimer and platelet were associated with DVT in patients with PICC. However, biomarkers such as APTT, fibrinogen, FDP, glucose, hemoglobin, glycated hemoglobin, INR, prothrombin time, prothrombin fragment 1.2, the thrombin–antithrombin complex, and WBC were not related to the development of DVT associated with PICC.

## 1. Introduction

The risk factors of deep venous thrombosis (DVT) associated with peripherally inserted central catheters (PICC) are extensively discussed in the literature. In contemporary clinical practice, there has been a substantial rise in the utilization of PICCs [1,2]. This surge in usage can be attributed to multiple factors, including the ease of insertion; the wide range of applications, such as medication administration and venous access; the perceived level of safety; and cost-effectiveness when compared to other central venous catheters (CVCs) [3].

However, PICC predispose patients to DVT by contemplating the Virchow’s triad. This prothrombotic effect can be attributed to the physical damage caused by the catheter (intimal injury), the impact of hemodynamic changes on the vessel wall (blood flow velocity), and alterations in the state of hypercoagulability (increased platelet levels) [3].

Studies have proposed the identification of plasma biomarkers as an additional predictor in the estimation of risk and early diagnosis of DVT related to PICC [2,3,4,5].

The limitations related to the diagnosis of DVT through imaging are associated with clinical manifestations such as localized pain, swelling, warmth and redness that may occur late, as well as the difficulty to access, via ultrasound, some deep veins of the upper extremity [6]. This situation compromises early diagnosis, delays treatment, prolongs hospitalization, and increases treatment costs [6,7].

Cohort studies have shown conflicting results regarding the association between biomarkers and DVT diagnosis [1,3]. Michigan researchers, when proposing a risk prediction score for thrombosis, identified that white blood cells >12,000 × 10^9^/μL were associated with DVT risk (OR 1.43; CI: 1.14–1.79; *p* = 0.0001), whereas platelets, international normalized ratio (INR), and hemoglobin had no statistically significant association [1]. On the other hand, Chinese researchers who proposed a nomogram for early DVT identification related to PICC showed no relationship with increased leukocytes. In contrast, platelets and D-dimer comprised the risk variables of this tool [3]. Subsequently, a study conducted by a different group of Chinese researchers showed no relationship among platelets, D-dimer, and thrombosis related to PICC [8].

From the evidence, we identified the lack of consensus among studies on biomarkers and their association with DVT development in patients with PICC. This systematic review with meta-analysis was conducted to fill this gap and sum up the results of studies involving PICC, biomarkers, and DVT. This study aimed to identify the association between the levels of potential biomarkers that reflect the activation of the blood system (D-dimer, FDP, activated partial thromboplastin time, fibrinogen, platelet count, plasminogen activator inhibitor 1), long-term vascular complications (blood glucose, glycated hemoglobin), inflammatory system (C-reactive protein, leukocyte count), and the occurrence of PICC-related DVT.

## 2. Methods

The Preferred Reporting Items for Systematic Reviews and Meta-Analysis (PRISMA) Statement [9] and Meta-analysis of Observational Studies in Epidemiology (MOOSE) [10] were followed as guidelines to perform and report this systematic review and meta-analysis. This systematic review is registered in the International Prospective Register for Systematic Review (PROSPERO) database under the number CRD42018108871.

### 2.1. Eligibility Criteria

Studies included in the analysis were required to include: (I) adult and pediatric patients, outpatient or admitted to clinical, surgical, or intensive care units that received PICC; (II) patients with PICC-related DVT and patients without PICC-related DVT as a comparator; and (III) at least one biomarker available from the following: activated partial thromboplastin time, D-dimer, fibrinogen, fibrinogen degradation product, glucose, glycated hemoglobin, hemoglobin, international normalized ratio, plasminogen, platelet, P-selectin, prostaglandin, protein c-reactive, prothrombin time, or white blood cells. No restrictions were made regarding gender, race, comorbidities, or clinical conditions.

We included observational studies (cohort, case–control studies, case reports, cross-sectional studies) and baseline data from quasi-experimental, randomized, or non-randomized clinical trials. Review articles that presented inclusion criteria had their quotations and references checked manually. We excluded studies according to these criteria: (1) conference abstracts, letters to the editor, or editorials; (2) research papers for repeated reports; and (3) studies not in English.

### 2.2. Information Sources

We searched electronic the databases Embase, Web of Science, Medline, Scopus, Cumulative Index to Nursing and Allied Health Literature (Cinahl), and Cochrane Central Register of Controlled Trials. We searched the ERIC database for gray literature. The search process was completed by December 2022.

### 2.3. Search Strategy

We used Boolean logic with keywords including “peripherally inserted central catheter”, “PICC”, “venous thromboembolism” and biomarkers: “D-Dimer”, “Hemoglobin”, “White Blood Cell”, “Leukocyte”, “Platelet Count”, “Prothrombin Time”, “P-Selectin”, “Neutrophil”, “Fibrinogen”, “Fibrinogen Degradation Product”, C-Reactive Protein”, “Lymphocyte”, and “Plasminogen Activator Inhibitor”. The complete search strategy used is shown in the electronic Supplementary Materials, Texts S1–S3.

### 2.4. Selection Process

The articles retrieved were uploaded to the Covidence systematic review software (www.covidence.org, accessed on 31 December 2022), and duplicated articles were excluded in the first step of the studies selection.

Both authors (P.C.C. and M.A.L.S.) independently evaluated the titles and abstracts of the studies according to the eligibility criteria. Any disagreements between the reviewers were first solved with a discussion and then, if necessary, through the arbitration of a third reviewer (E.R.R.S. or P.M.B.). All abstracts that did not provide enough information regarding the inclusion and exclusion criteria were evaluated in the full text.

A manual search (i.e., reference lists and citation searching) of review studies fulfilling the eligibility criteria was also carried out. Finally, all studies that met the eligibility criteria in full text were included in the data extraction process. The corresponding author was contacted in case it was necessary to obtain the data not included in the published report.

### 2.5. Data Collection Process

The extracted data were registered in a standardized document prepared by the researchers on the Covidence website. Data were independently extracted in duplicates (P.C.C. and M.A.L.S.) using a standardized and tested data extraction spreadsheet. Missing data were requested from the study authors. Any disagreements between reviewers were first solved through discussion and then, if necessary, the arbitration of a third reviewer (P.M.B. or E.R.R.S.).

### 2.6. Data Items

The following information was extracted from included studies:

- Article: author, title, year of publication, study design.
- Sample characteristics: sample number, the incidence of thrombosis, mean (average) age of the patients, pathologies, hospitalized or outpatient clinic.
- Diagnosis of DVT in patients with PICC: PICC-related DVT according to the diagnosis presented by authors.
- PICC characteristics: indication, the duration of PICC use (or time to thrombosis).
- Biomarkers: Means ± standard deviations or narrative syntheses were extracted for continuous variables related to blood biomarker evaluation.

### 2.7. Study Risk of Bias Assessment

The risk-of-bias assessment in the included studies was performed by two authors (P.C.C., M.A.L.S.) independently, using the Newcastle–Ottawa Quality Assessment Scale, which uses a star system to evaluate the quality of a study in three domains: the selection of study groups; group comparability; and results verification. The studies that received a star in every domain were considered high quality [11]. Publication bias was assessed using a contour-enhanced funnel plot of each trial’s effect size against the standard error of the estimate.

### 2.8. Synthesis Methods

We aimed to synthesize the results from the included studies, structured around the type of outcome. Meta-analysis was conducted using RevMan software (Cochrane Review Manager, v5.3). Blood biomarker outcomes were expressed as mean difference (MD) and 95% confidence interval (CI) or standardized mean difference (SMD), used as a summary statistic in meta-analysis when the studies all assess the same outcome but measured in a variety of ways, between DVT and non-DVT groups. We pooled the results using a random effects meta-analysis. A *p*-value of less than 0.05 was considered statistically significant.

The statistical heterogeneity of the effect among studies was assessed using the chi-squared test and I-squared statistic. According to the Cochrane Handbook for Systematic Reviews of Interventions, we considered an I-squared (I^2^) value greater than 70% indicative of possible substantial heterogeneity with a threshold *p*-value of 0.1 as statistically significant. A meta-regression was conducted to further investigate the heterogeneity between studies. Univariate meta-regression models were performed in STATA software (v20) to assess clinical and methodological variables associated with activated partial thromboplastin time, D-dimer, fibrinogen, and international normalized ratio, age, and male gender, based on R2 values and statistical significances of *p* < 0.05.

This review also summarized the available results from the literature about biomarkers and DVT and presented them according to the synthesis without meta-analysis (SWiM)-reporting guidelines [12] due to qualitative reports in some original studies. To carry out this evaluation, the results of the studies were summed up in a chart, according to biomarkers, with the data on the population of the study and the summary findings of biomarker levels and TE rates, as well as TE risks, namely the hazard ratio (HR) and odds ratio (OR).

## 3. Results

### 3.1. Study Selection

**Figure 1:**
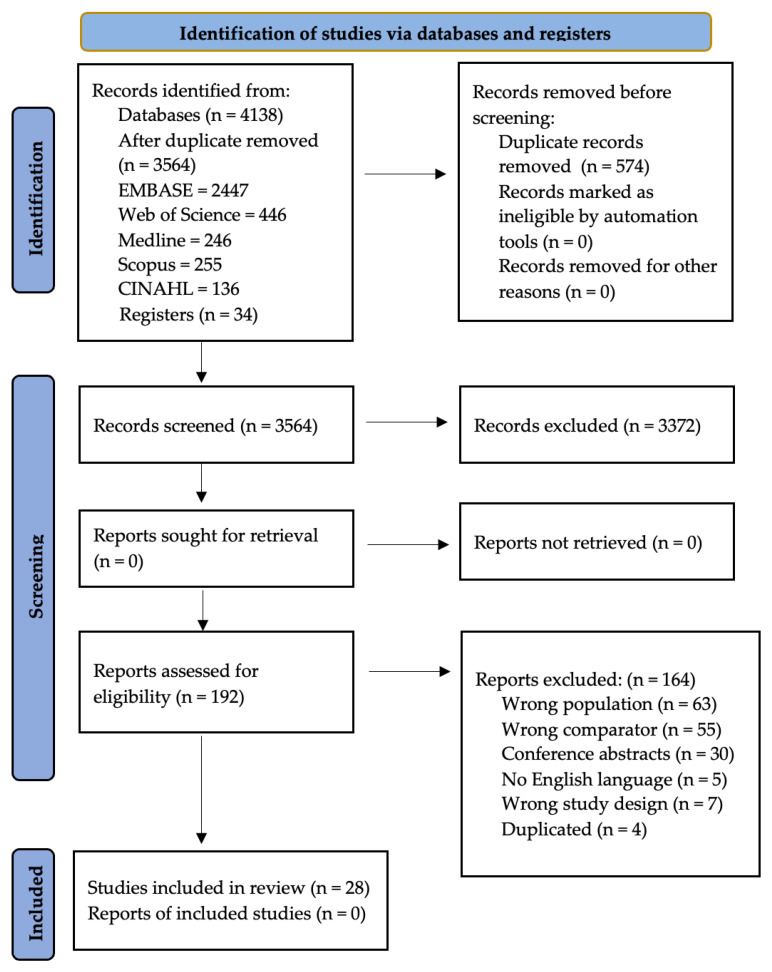
PRISMA 2020 flow diagram for new systematic reviews, which included searches of databases and registers only.

The search in the databases identified 4138 potentially eligible studies. After the removal of duplicates, 3564 studies were selected for review of titles and abstracts: Embase = 2447, Web of Science = 446, Medline = 246, Scopus = 255, Cinahl = 136, Cochrane Central Register of Controlled Trials = 0, and ERIC = 0. In addition, 34 titles and abstracts were evaluated for inclusion through reading review articles. A total of 192 full-text articles retained from this stage were reassessed, of which 28 were included. A detailed flowchart showing the study selection process is presented in Figure 1.

### 3.2. Study Characteristics

**Figure 2:**
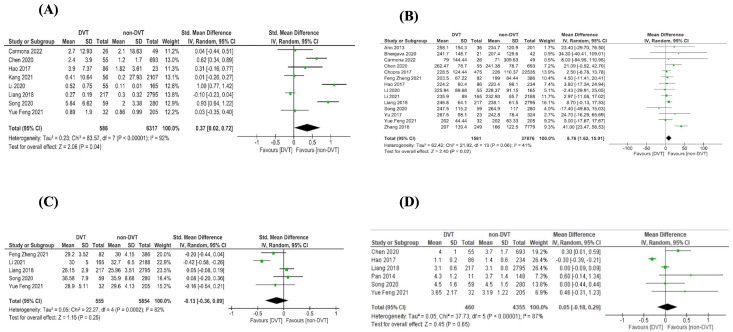
Forest plot. (**A**) D-dimer [3,8,13,17,18,21,34,36]; (**B**) platelets [1,3,8,13,14,15,16,18,19,21,22,34,35,36]; (**C**) activated partial thromboplastin time [8,13,18,21]; (**D**) fibrinogen [3,8,13,20,21,36].

**Figure 3:**
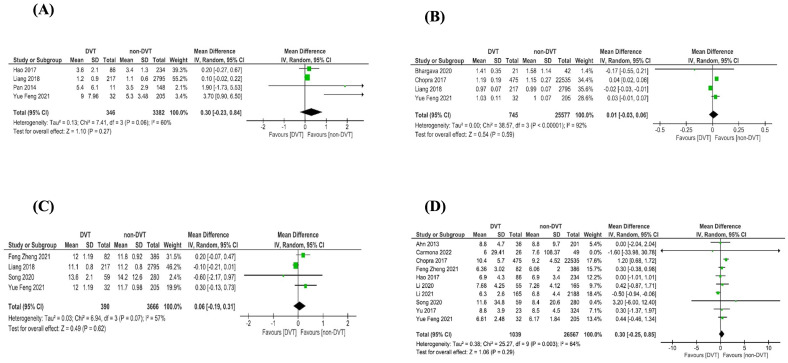
Forest plot. (**A**) Fibrinogen degradation product [3,8,13,20]; (**B**) international normalized ratio [1,8,13,16]; (**C**) prothrombin time [8,13,21,35]; (**D**) white blood cell [1,3,13,15,18,19,21,22,34,35].

In total, this review included twelve biomarkers in twenty-eight articles: twelve articles [3,8,13,14,15,16,17,18,19,20,21,22] were analyzed via meta-analysis; twelve articles [5,23,24,25,26,27,28,29,30,31,32,33] were analyzed qualitatively; and four articles [1,34,35,36] were analyzed via meta-analysis and qualitatively. Table S1 describes the characteristics of the studies included in the meta-analysis, and Figure 2 and Figure 3 illustrate the meta-analysis results. Table S2 shows the qualitatively analyzed biomarkers.

The green squares represent the standard mean difference or mean difference (MD) of biomarkers between groups (DVT and non-DVT). The horizontal lines represent the 95% confidence intervals (CI). The black diamond represents the overall effect estimate of the meta-analysis.

The green squares represent the mean difference (MD) of biomarkers between groups (DVT and non-DVT). The horizontal lines represent the 95% confidence intervals (CI). The black diamond represents the overall effect estimate of the meta-analysis.

In all the studies, a final assessment was carried out and the following outcomes were reported: platelets (23 articles) [1,3,8,13,14,15,16,18,19,21,22,23,25,26,27,29,30,31,32,33,34,35,36], followed by white cells (15 articles) [1,3,13,15,18,19,21,22,24,27,29,30,31,34,35], D-dimers (13 articles) [3,8,13,17,19,21,23,26,27,28,34,35,36], fibrinogen (10 articles) [3,8,13,20,21,23,25,26,32,36], APTT (6 articles) [8,13,19,21,23,35], prothrombin time (6 articles) [8,13,21,25,32,35], hemoglobin (5 articles) [1,23,27,29,30], fibrinogen degradation product (4 articles) [3,8,13,20], INR (4 articles) [1,8,13,16], HbA1c (1 article) [5], prothrombin fragment 1.2 (1 article) [34], and the thrombin–antithrombin complex (1 article) [34].

Out of the 28 studies, 20 included the oncological and hematological population [8,13,14,15,18,19,22,23,24,25,27,29,30,31,32,33,34,35,36] and seven studies included hospitalized patients [1,3,5,16,17,20,28]. Out of the 28 studies, only two included children and adolescents [26,34].

Regarding the study design, 18 were retrospective [3,8,14,15,16,17,20,21,22,23,24,25,28,30,31,32,33,36] and 10 were prospective [1,5,13,18,19,26,27,29,34,35].

### 3.3. Risk of Bias in Studies and Publication Bias Assessment

The Newcastle–Ottawa Scale was used to evaluate the quality of the studies. All studies had a Newcastle–Ottawa score of 9/9 (range from 1 to 9), which is considered high quality (Table S3).

The possibility of publication bias was evaluated using a funnel plot for platelet level (Figure S1). The points for the missing studies would be found at the bottom of the plot, but the plot is symmetrical and publication bias is unlikely to be present.

### 3.4. Results of Syntheses

The data from the meta-analysis on the impact of DVT on biomarkers are presented in Figure 2 and Figure 3.

### 3.5. D-Dimer

Thirteen articles included D-dimers in the thrombosis analysis related to PICC, eight in the meta-analysis and five qualitatively.

According to meta-analysis [3,8,21] (Figure 2A), PICC related to DVT is associated with higher D-dimers (0.37 μg/mL, 95% CI 0.02, 0.72; *p* = 0.04, I^2^ = 92%; *p* for heterogeneity < 0.00001). This analysis included seven studies involving oncological patients [8,13,17,18,21,34,36] and one involving hospitalized patients [3]. Seven studies were conducted on an adult population [3,8,13,17,18,21,37] and one on children and adolescents [26]. Meta-regression analyses applied to studies included in D-dimer analyses indicated that age (adjusted R2 = 7.57%; *p* = 0.2387) and male gender (adjusted R2 = 0%; *p* = 0.4342) were not associated with differences among studies (Table S4).

For qualitative analysis, five studies were included (Table S2). One study conducted on 370 patients with non-Hodgkin’s lymphoma showed that increased D-dimer is a factor contributing to the occurrence of PICC-related thrombosis (thrombosis group vs. non-thrombosis; *p* < 0.001) [27]. Another retrospective cohort study on 1312 breast cancer patients showed that D-dimer was considered a significant PICC-RVT predictive factor (OR 3.673; CI 95% 1.698–7.946; *p* = 0.001) [23]. However, three studies showed no association between D-dimer- and PICC-related DVT [26,28,32].

### 3.6. Platelet Count

In this review, 23 articles included platelets in the thrombosis analysis related to PICC. According to fourteen articles [2,3,8,14,15,16,18,19,21,22,34,35,36] included in the meta-analysis (Figure 2B), PICC related to DVT is associated with higher platelets (8.76 × 10^9^/L, 95% CI 1.62, 15.91; *p* = 0.02, I^2^ = 41%; *p* for heterogeneity = 0.06). This analysis included eleven studies of onco-hematological populations [8,13,14,15,18,19,21,22,34,35,36], two of hospitalized patients [1,3], and one of critical patients [16]. With the exception of one study with children and adolescents [34], all studies involved adults.

In the qualitative analysis (Table S2), eight articles [25,26,27,29,30,31,32,33] showed no statistically significant results. The patients were mainly diagnosed with cancer, specifically lung cancer, breast cancer, and non-Hodgkin’s lymphoma. In one retrospective study of breast cancer patients (sample size = 1312), platelets were considered a significant PICC-RVT predictive factor (OR: 3.783, CI: 1.756–8.149, *p* = 0.001) [23].

### 3.7. Activated Partial Thromboplastin Time (APTT)

Six articles included APTT in the thrombosis analysis related to PICC, five in the meta-analyses and one qualitatively.

According to meta-analysis [8,13,19,21,35] (Figure 2C), APTT was not associated with PICC related to DVT (−0.13 s, 95%CI −0.36, 0.09; *p* = 0.25, I^2^ = 82%; *p* for heterogeneity = 0.0002). This analysis included adults with oncological diseases. Meta-regression analyses applied to studies included in APTT analyses indicated that age (adjusted R2 = 0%; *p* = 0.6583) and male gender (adjusted R2 = 0%; *p* = 0.6660) were not associated with differences among studies (Table S4).

We analyzed qualitatively one retrospective article conducted on a breast cancer population (sample size = 1312) [23] (Table S2), and they considered APTT to be a predicted factor to thrombosis related to PICC (OR 7.112; CI 1.278–39.571; *p* = 0.025).

### 3.8. Fibrinogen

Seven articles included fibrinogen in the thrombosis analysis related to PICC. Four were analyzed in the meta-analysis and three were qualitative.

According to meta-analysis [3,8,13,20,21,36] (Figure 2D), fibrinogen was not associated with PICC related to DVT (0.05 g/L, 95%CI −0.18, 0.29; *p* = 0.65, I^2^ = 87%; *p* for heterogeneity < 0.00001). This analysis included adult patients, four studies on oncological patients [8,13,21,36] and two on hospitalized patients [3,20]. Meta-regression analyses applied in studies included in fibrinogen analyses indicated that age (adjusted R2 = 0%; *p* = 0.4315) and male gender (adjusted R2 = 0%; *p* = 0.2517) were not associated with differences among studies (Table S4).

In the qualitative analysis (Table S2), two studies of oncological adult patients with lung cancer [25,32] associated higher fibrinogen levels with PICC–DVT. However, a study of children and adolescents with oncologic diseases [26] and a study of breast cancer patients [23] showed no association between fibrinogen and PICC related to DVT.

### 3.9. Fibrinogen Degradation Product (FDP)

Four articles were included in the meta-analysis [3,8,13,21] (Figure 3A), and FDP was not associated with PICC related to DVT (0.30 mcg/mL, 95% CI −0.23, 0.84; *p* = 0.027, I^2^ = 60%; *p* for heterogeneity = 0.06). To evaluate the FDB biomarker and DVT risks, four studies were included in the meta-analysis: a study of 320 hospitalized patients [3], a study of 3012 patients diagnosed with non-metastatic nasopharyngeal carcinoma [8], a study of 237 patients diagnosed with lung cancer [13], and a study of 2313 hospitalized patients [20]. All studies were conducted in an adult population in China between the years of 2014 to 2021.

### 3.10. International Normalized Ratio (INR)

Four articles included INR in the thrombosis analysis related to PICC. According to meta-analysis (Figure 3B), INR was not associated with PICC related to DVT (0.01 s, 95%CI −0.03, 0.06; *p* = 0.59, I^2^ = 92%; *p* for heterogeneity < 0.00001). Meta-regression analyses applied in studies included in the INR analyses indicated that age (adjusted R2 = 0%; *p* = 0.9408) and male gender (adjusted R2 = 44.27%; *p* = 0.1215) were not associated with differences among studies (Table S4). This analysis included two studies of patients with oncological diseases [8,13], one of critical patients [16], and one of hospitalized patients [1].

### 3.11. Prothrombin Time

Six articles included prothrombin time in the thrombosis analysis related to PICC. Four were analyzed in the meta-analysis and two were qualitative.

According to meta-analysis (Figure 3C), prothrombin time was not associated with PICC related to DVT (0.06 s, 95%CI −0.19, 0.31; *p* = 0.62, I^2^ = 57%; *p* for heterogeneity = 0.07). This analysis included four studies of oncological adult patients [8,13,21,35].

Two articles were analyzed [25,32] qualitatively (Table S2) and showed no association between prothrombin time PICC related to DVT. The studies analyzed were retrospective and conducted in adults and oncologic patients.

### 3.12. White Blood Cells (WBCs)

For WBCs, fifteen articles included white cells in the thrombosis analysis related to PICC, ten in were meta-analyses and five were qualitative.

According to the meta-analysis [1,3,13,15,18,19,21,22,34,35] (Figure 3D), WBC count was not associated with PICC related to DVT (0.30 × 10^9^/L, 95% CI −0.25, 0.88; *p* = 0.29, I^2^ = 64%; *p* for heterogeneity = 0.003). This analysis included eight articles studying oncological and hematological populations [13,15,18,19,21,22,34,35] and two studying hospitalized patients [1,3]. Except one study that involved children and adolescents [26], all studies encompassed adults.

In the qualitative analysis (Table S2), four articles on oncological patients [24,27,30,31] showed no statistically significant results. However, a study involving cancer patients undergoing chemotherapy [29] showed that WBC (>11.4 × 10^9^/L) was a significant risk factor for thrombosis.

### 3.13. Blood Glucose

This biomarker was analyzed in a case–control study conducted on hospitalized patients (Table S2) [5], and it was demonstrated that the mean glucose at admission was significantly higher for cases of PICC related to DVT than for controls (176.9 mg/dL vs. 148.9 mg/dL, *p* = 0.002).

### 3.14. Glycated Hemoglobin (HbA1c)

This biomarker was analyzed in a case–control study, published in 2018, conducted in hospitalized patients, comparing patients with PICC-related DVT to patients with PICCs who did not develop DVT (Table S2) [5]. PICC-related DVT cases were more likely to be diabetic, but it no association between HbA1c and thrombosis related to PICC was revealed (DVT 7.3 (5.4–11.1, SD = 1.9) vs. non-DVT 7.6 (4.3–14.7, SD = 2.4); *p* = 0.84).

### 3.15. Hemoglobin

We analyzed qualitatively five studies that included hemoglobin in the thrombosis analysis related to PICC (Table S2).

One study of 23,010 adult patients admitted to a general medicine ward or intensive care unit showed that higher levels of hemoglobin were associated with PICC-related thrombosis (non-DVT 10.20 (8.80–11.70) vs. DVT 9.80 (8.60–11.50); *p* < 0.01) [1]. Likewise, a study conducted on 370 patients with non-Hodgkin’s lymphoma showed statistically significant results (thrombosis group vs. non-thrombosis group; *p* < 0.001)—being higher in patients with DVT [27]. However, three studies on oncological patients showed no association between hemoglobin and thrombosis related to PICC [23,29,30].

### 3.16. Prothrombin Fragment 1.2 and Thrombin–Antithrombin Complex

The prothrombin fragment 1.2 and the thrombin–antithrombin complex biomarkers were included in one study of 75 children and adolescents, aged 1–21 years, with acute lymphoblastic leukemia (Table S2). According to this prospective cohort study, at the baseline, the prothrombin fragment biomarker was not associated with PICC-related thrombosis (DVT vs. non-DVT 377 pmol/L vs. 316 pmol/L; *p* = 0.16). Throughout the follow-up period, prothrombin fragment 1.2 values reached their highest point in all participants on day 14 and gradually declined until day 28. This biomarker exhibited significantly higher levels in participants diagnosed with DVT compared to those without DVT (OR 1.31, 95% CI: 1.25–1.37) at all time points, considering age, sex, and risk group adjustments.

The data of thrombin-antithrombin complexes did not exhibit statistical significance (*p* = 0.32) at baseline when comparing DVT and non-DVT. However, during the 28-day follow-up period, this biomarker was significantly elevated in participants diagnosed with DVT (OR 1.34, 95% CI: 1.32–1.38).

## 4. Discussion

This is the first systematic review with a meta-analysis that aimed to compile evidence on the association of biomarkers and DVT in patients using PICC. The collection of biomarkers for detecting DVT occurrence before the development of clinical symptoms would allow immediate treatment and, consequently, reduce complications, such as scarring and stenosis in the veins [38], as well as life-threatening events, such as pulmonary embolism [39]. Moreover, identifying biomarkers associated with thrombosis could contribute to stratifying the DVT risk in patients with PICC, as suggested by Chinese [3] and Michigan [1] researchers. Overall, the evidence generated by this review indicates that D-dimer and platelets can be considered DVT biomarkers associated with PICC. D-dimer is a soluble fibrin degradation product and exhibits properties as a biological marker of hemostatic abnormalities as well as an indicator of intravascular thrombosis. D-dimer has been extensively investigated for excluding the diagnosis of venous thromboembolism. The limitations of the assay include D-dimer elevation in a constellation of clinical scenarios (age, pregnancy, and cancer) and a lack of clinical standardization [40]. Platelets are defined as playing a vital role in homeostasis and thrombosis, but this role has expanded to include inflammation, cancer progression, and metastasis [41]. Despite our findings, the D-dimer and platelets biomarker were studied mostly in onco-hematological and hospitalized adult patients, and the validation of this biomarker in other populations is necessary before recommending its use.

In this meta-analysis, the level of APTT, fibrinogen, FDP, INR, prothrombin time, and white blood cells were not associated with thrombosis related to PICC. Similarly, the qualitative analysis of APTT, fibrinogen, glucose, hemoglobin, HbA1c, prothrombin fragment 1,2, prothrombin time, thrombin–antithrombin complex, and white blood cell did not show clinically relevant results to associate thrombosis related to PICC. From the results of this review, it is impossible to infer why these biomarkers were not associated with thrombosis related to PICC. The development process of DVT is complex and multifactorial and may interfere in patient-related conditions, such as cancer diagnosis, diabetes, and obesity [42]; the use of medications, such as enoxaparin [43]; chemotherapy [42]; issues related to PICC, such as electrocardiogram use [44]; and ultrasound use for guiding the insertion [45], among others.

Most of the studies included populations at higher risk for thrombosis, predominating oncological patients. Thus, when we try to explain physiological changes in coagulation in patients with PICC, considering that these patients already show alterations resulting from the underlying disease and its treatments, the results may have limitations. The relationship between cancer and thrombosis was established centuries ago [46]. DVT may reflect tumor biology; for instance, the activation of the coagulation cascade and thrombin generation is often cited as a mechanism by which tumor propagation can occur [47]. Vascular access devices often triggers thrombotic events in cancer patients [15,48]. In addition, chemotherapy regimens can cause severe damage to the vascular intima and induce phlebitis and local inflammation due to the infusion of chemo-agents.

Therefore, chemotherapy is considered a risk factor for thrombosis. Chronic inflammation induced by the infusion of the chemotherapy agent can cause vasoconstriction and alter hemodynamics, facilitating the occurrence of thrombosis. Moreover, chemotherapy regimens can alter the local pH of the blood, which can directly affect the venous endothelium and promote the formation of thrombi [15].

The evolution of knowledge and the development of new technologies concerning the use of vascular access devices allowed the improvement of PICC indications [49,50]. Clinical outcomes such as DVT, which previously contraindicated the use of PICC in subgroups of patients, such as oncological and critical [51], were questioned by systematic reviews via meta-analyses [49,50]. These two reviews indicated that PICC insertion adhering to best practices and adding technology reduced the risk of complications such as DVT. Adding technologies, such as ultrasound-guided PICC insertion [52]; electrocardiograms for the confirmation of the PICC tip [53]; catheter caliber selection suitable for vessel lumen, occupying up to 45% of its light [54,55]; and PICC venipuncture in the zone insertion method (ZIM Zone) [56], are associated with reduced incidence of DVT.

Identifying biomarkers in patients with PICC is an opportunity to contribute to the early diagnosis of DVT [6,7] and to identify patients at higher risk of developing this complication [1,3], making care practice safer for patients with PICC.

## 5. Limitations

Our systematic review with meta-analysis shows some limitations, such as the identification of predominant studies conducted in samples of oncological adult patients subjected to chemotherapy, developed in single centers with retrospective data, small sample sizes, and especially with different biomarkers that made the composite analysis fragile in meta-analysis. It was also not possible to analyze the interference of medications, such as anticoagulants and antiplatelet drugs at the levels of biomarkers, due to the absence of information provided by the authors. However, our study adds to the knowledge on this topic as the first meta-analysis on biomarkers related to PICC-DVT. Thus, our study may serve as support for more robust studies that aim to elucidate the relationship between biomarkers and DVT in patients with PICC.

## 6. Conclusions

Therefore, it is evident that, via meta-analysis, higher levels of D-dimers and platelets are associated with DVT in patients with PICC. APTT, fibrinogen, FDP, glucose, hemoglobin, HbA1c, INR, prothrombin time, and white blood cell prothrombin fragment 1,2, prothrombin time, the thrombin–antithrombin complex, and white blood cell were not related to the development of DVT associated with PICC. This review highlights the need for further studies to elucidate the action of the D-dimer and platelet biomarkers in other populations.

## Data Availability

All data generated or analyzed during this study are included in this published article (and its Supplementary Materials).

## Supplementary Materials

The following supporting information can be downloaded at: https://www.mdpi.com/article/10.3390/jcm12134480/s1, Text S1: Literature search strategy used for the PubMed database; Text S2: Literature search strategy used for the Embase database; Text S3: Literature search strategy used for the Web of Science database; Figure S1: Funnel plot for platelets; Table S1: Characteristics of included studies in meta-analysis; Table S2: Summary findings from included studies in qualitative analysis listed by biomarker; Table S3: Quality of included studies using Newcastle–Ottawa scale for non-randomized studies; Table S4: Meta-regression with activated partial thromboplastin time, D-dimer, fibrinogen and international normalized ratio, age, and male gender.
