# Supplementary material for: Biomarkers Associated with Thrombosis in Patients with Peripherally Inserted Central Catheter: A Systematic Review and Meta-Analysis
Source: J Clin Med. 2023 Jul 4;12(13):4480. doi: 10.3390/jcm12134480 (PMC10342538; doi:10.3390/jcm12134480)
Supplement: Supplementary file 1 [file jcm-12-04480-s001.zip › jcm-2363003-supplementary.pdf]

Supplementary material

ESM Text S1: Literature search strategy used for the pubmed database

Biomarkers[mesh] OR biomarker[tw] OR biomarkers[tw] OR marker[tw] OR markers[tw] OR endpoint[tw] OR "end point"[tw] OR endpoints[tw] OR "end points"[tw]

OR

D-dimer[tw] OR haemoglobin[tw] OR hemoglobin[tw] OR "white cell"[tw] leukocyte[tw] OR leukocytosis[tw] OR "platelet count"[tw] OR "platelet counts"[tw] OR "clotting factor"[tw] OR "clotting factors"[tw] OR prothrombin[tw] OR P-selectin[tw] OR "blood count parameters"[tw] OR khorana[tw] OR neutrophil[tw] OR monocyte[tw] OR fibrin[tw] OR thrombopoietin[tw] OR "thrombin generation potential"[tw] OR fibrinogen[tw] OR "factor XIII"[tw] OR FXIII[tw] OR "factor VIII"[tw] OR FVIII[tw] OR c-reactive[tw] OR CRP[tiab] OR "factor V Leiden"[tw] OR FVL[tiab] OR RAM[tiab] OR WBC[tiab] OR cytokine[tw] OR cytokines[tw] OR chemokine[tw] OR chemokines[tw] OR "immune-related effectors"[tw] OR "acute-phase proteins"[tw] OR "reactive oxygen"[tw] OR "reactive nitrogen"[tw] OR prostaglandin[tw] OR prostaglandins[tw] OR "cyclooxygenase-related factors"[tw] OR "transcription factor"[tw] OR "transcription factors"[tw] OR "growth factor"[tw] OR "growth factors"[tw] OR mGPS[tw] OR albumin[tw] OR NLR[tiab] OR lymphocyte[tw] OR lymphocytes[tw] OR PLR[tw] OR SAA[tw] OR ROS[tw] OR RNS[tw] OR 3-Nitrotyrosine[tw] OR 8-oxodg[tw] OR 8-OHdG[tw] OR MDA[tw] OR HNE[tw] OR COX2[tw] OR NF-κB[tw] OR STAT3[tw] OR "plasminogen activator inhibitor 1"[tw] OR PAI-1[tw]

AND

Thromboembolism[mesh] OR Thrombosis[mesh] OR "Pulmonary Embolism"[mesh] OR thromboembolism[tw] OR thrombosis[tw] OR thrombus[tw] OR TE[tiab] OR DVT[tiab] OR VTE[tiab] OR thrombii[tiab] OR clot[tiab] OR clots[tiab] OR clotting[tiab] or embolism[tiab] OR thrombotic[tiab] OR thrombolic[tw] OR embolic[tw]

AND

(PICC[tw] OR PICCs[tw] OR “peripherally inserted central catheter”[tw] OR “peripherally inserted central catheters”[tw] OR “peripheral venous catheterization”[tw] OR “peripheral venous catheter”[tw] OR “peripheral venous catheters”[tw] OR "Catheterization, Peripheral"[Mesh] OR "Catheters, Indwelling"[Mesh])

ESM Text S2: Literature search strategy used for the Embase database

('biological marker'/exp OR 'biological marker' OR 'biological markers' OR 'biomarker' OR 'biomarkers' OR 'marker, biological' OR 'd dimer'/exp OR 'd dimer' OR 'crosslinked fibrin degradation product' OR 'fibrin degradation product d dimer' OR 'hemoglobin'/exp OR 'hb' OR 'ferrohaemoglobin' OR 'ferrohemoglobin' OR 'free haemoglobin' OR 'free hemoglobin' OR 'haemoglobin' OR 'haemoglobine' OR 'haemoglobins' OR 'hemoglobin' OR 'hemoglobine' OR 'hemoglobins' OR 'hemoglobulin' OR 'trm 645' OR 'trm645' OR 'unstable haemoglobin' OR 'unstable hemoglobin' OR 'leukocyte'/exp OR 'human leucocyte' OR 'human leukocyte' OR 'leucocyte' OR 'leucocytes' OR 'leukocyte' OR 'leukocytes' OR 'peripheral blood leucocyte' OR 'peripheral blood leukocyte' OR 'peripheral leucocyte' OR 'peripheral leukocyte' OR 'wbc' OR 'white blood cell' OR 'white blood corpuscle' OR 'white cell' OR 'platelet count'/exp OR 'blood platelet count' OR 'count, blood platelet' OR 'count, thrombocytic' OR 'platelet count' OR 'platelet counting'OR 'platelet number' OR 'thrombocyte count' OR 'thrombocyte counting' OR 'thrombocyte number' OR 'blood clotting factor'/exp OR 'antihaemophilic agent' OR 'antihaemophilic agents' OR 'antihemophilic agent' OR 'antihemophilic agents' OR 'blood clotting factor' OR 'blood clotting factor activity' OR 'blood clotting factors' OR 'blood coagulating factor' OR 'blood coagulation factor' OR 'blood coagulation factors' OR 'clot promoting factor' OR 'clotting factor' OR 'clotting factor activity' OR 'clotting factor level' OR 'clotting factor, nonclassified' OR 'coagulation factor' OR 'congenital clotting factor' OR 'nonclassified blood clotting factor' OR 'prothrombin'/exp OR 'blood clotting factor 2' OR 'blood clotting factor ii' OR 'bovine prothrombin' OR 'carboxylated prothrombin' OR 'carboxyprothrombin' OR 'clotting factor 2' OR 'coagulation factor ii'OR 'factor 2' OR 'factor ii' OR 'plasma prothrombin' OR 'plasmozyme' OR 'proserozyme' OR 'prothrombin' OR 'prothrombin bovine' OR 'prothrombin index' OR 'prothrombin, carboxylated' OR 'prothrombine' OR 'serozyme' OR 'thrombinogen' OR 'thrombogen' O R 'padgem protein'/exp OR 'cd62 antigen' OR 'cd62p antigen' OR 'gmp 140' OR 'p selectin' OR 'padgem protein' OR 'alpha granule membrane protein 140' OR 'antigen cd62p' OR 'gmp140' OR 'granule membrane protein 140' OR 'granule membrane protein gmp 140' OR 'p-selectin' OR 'platelet activation dependent granule to external

membrane glycoprotein' OR 'platelet alpha granule membrane protein 140' OR 'selectin p' OR 'blood cell count'/exp OR 'blood cell count' OR 'blood count' OR 'complete blood count' OR khorana OR 'neutrophil'/exp OR 'granulocyte, neutrophil' OR 'leucocyte, neutrophil'OR 'leukocyte,
neutrophil' OR 'neutrocyte' OR 'neutrocytes' OR 'neutrophil' OR 'neutrophil granulocyte' OR 'neutrophil leucocyte' OR 'neutrophil leukocyte' OR 'neutrophilic granulocyte' OR 'neutrophilic leucocyte' OR 'neutrophilic
leukocyte' OR 'neutrophils' OR 'pmn granulocyte' OR 'pmn leucocyte' OR 'pmn leukocyte' OR 'pmn neutrophil' OR 'polymorphonuclear
granulocyte' OR 'polymorphonuclear leucocyte' OR 'polymorphonuclear
leukocyte' OR 'polymorphonuclear neutrophil' OR 'polymorphous
leucocyte' OR 'polymorphous leukocyte' OR 'polynuclear leucocyte' OR 'polynuclear leukocyte' OR 'monocyte'/exp OR 'monocyte' OR 'monocytes' OR 'monocytes, activated killer' OR fibrinOR 'thrombopoietin'/exp OR 'mgdf' OR 'megakaryocyte growth and development factor' OR 'thrombocytopoiesis stimulating
factor' OR 'thrombopoietin' OR 'thrombin generation potential' OR 'fibrinogen'/exp
OR 'blood clotting factor 1' OR 'blood clotting factor i' OR 'clottagen' OR 'clotting factor 1' OR 'factor 1' OR 'factor i' OR 'fibclot' OR 'fibrinogen' OR 'fibryga' OR 'human fibrinogen' OR 'blood clotting factor 13'/exp OR 'blood clotting factor 13' OR 'blood clotting factor xiii' OR 'blood coagulation factor xiii' OR 'bloodclotting factor
13' OR 'clotting factor 13' OR 'clotting factor xiii' OR 'duckert factor' OR 'factor
13' OR 'factor xiii' OR 'fibrin stabilising factor' OR 'fibrin stabilizing factor'OR 'plasma transglutaminase' OR 'thrombocyte factor xiii' OR fxiii OR 'c reactive protein'/exp OR 'c reactive protein' OR 'c reaction protein' OR 'c-reactive protein' OR 'creactive
protein' OR 'crp' OR 'protein, c reactive' OR 'serum c reactive protein' OR 'blood clotting factor 5 leiden'/exp OR 'blood clotting factor 5 leiden' OR 'blood clotting factor v leiden' OR 'factor v leiden' OR ram OR 'cytokine'/exp
OR 'cytokine' OR 'cytokines' OR 'interleukin' OR 'chemokine'/exp
OR 'chemokine' OR 'chemokines' OR 'chemotactic cytokine' OR 'immune-related effectors' OR 'acute phase protein'/exp OR 'acute phase globulin' OR 'acute phase glycoprotein' OR 'acute phase plasma protein'OR 'acute phase protein' OR 'acute phase proteins' OR 'acute phase reactant' OR 'acute phase response protein' OR 'acute-phase proteins' OR 'globulin, acute phase' OR 'glycoprotein, acute phase' OR 'plasma protein, acute phase' OR 'protein, acute phase' OR 'reactive oxygen metabolite'/exp OR 'reactive

oxygen metabolite' OR 'reactive oxygen species' OR 'reactive nitrogen' OR 'prostaglandin'/exp OR 'prostaglandin' OR 'prostaglandin like activity' OR 'prostaglandin like material' OR 'prostaglandin like substance' OR 'prostaglandins' OR 'cyclooxygenase-related factors' OR 'transcription factor'/exp OR 'pol1 transcription initiation complex proteins' OR 'transcription factor'OR 'transcription factors' OR 'growth factor'/exp OR 'growing factor' OR 'growth factor' OR 'growth factor, epithelial cell' OR mgps OR 'albumin'/exp OR 'albumen' OR 'albumin'OR 'albumin secretion' OR 'albumin variant' OR 'liquid albumin' OR nlr OR 'lymphocyte'/exp OR 'blood lymphocyte' OR 'f1 lymphocyte' OR 'immune competent cell' OR 'immune lymphocyte' OR 'immune lymphoid cell' OR 'immunocyte' OR 'large lymphocyte' OR 'lymph cell' OR 'lymphocyte' OR 'lymphocyte f1' OR 'lymphocyte kinetics' OR 'lymphocyte, immune' OR 'lymphocytes' OR 'memory lymphocyte' OR 'small lymphocyte' OR 'platelet lymphocyte ratio'/exp OR 'plr (lymphocyte)' OR 'platelet lymphocyte ratio' OR 'platelet to lymphocyte ratio' OR 'platelet/lymphocyte ratio' OR 'thrombocyte lymphocyte ratio' OR saa OR ros OR rns OR '3 nitrotyrosine'/exp OR '3 nitrotyrosine' OR 'nitrotyrosine' OR '8 oxodgtpase'/exp OR '8 ohdg' OR mda OR hne OR cox2 OR 'nf κb' OR stat3 OR 'plasminogen activator inhibitor 1'/exp OR 'endothelial type plasminogen activator inhibitor' OR 'mesosecrin' OR 'plasminogen activator inhibitor 1' OR 'plasminogen activator inhibitor type 1' OR 'pai 1') AND ('thromboembolism'/exp OR 'cerebral embolism and thrombosis' OR 'embolism and thrombosis' OR 'embolism, thrombo' OR 'intracranial embolism and thrombosis' OR 'thrombo embolic disease' OR 'thrombo embolism' OR 'thromboembolic' OR 'thromboembolic complication' OR 'thromboembolic disease' OR 'thromboembolic process' OR 'thromboembolism' OR 'thromboembolus'OR 'thromboemboly' OR 'thrombosis'/exp OR 'acute thrombosis' OR 'arm thrombosis' OR 'atherothrombosis' OR 'rethrombosis' OR 'sclerothrombosis' OR 'thromboobliterative disease' OR 'thromboocclusive disease' OR 'thrombosis' OR 'thrombosis induction' OR 'thrombotic disease' OR 'thrombotic occlusion' OR 'lung embolism'/exp OR 'chronic lung embolism' OR 'embolism, lung' OR 'lung embolism' OR 'lung embolization' OR 'lung embolus' OR 'lung embolus recurrence' OR 'lung emboly'OR 'lung microembolism' OR 'lung microembolization' OR 'lung microembolus' OR 'lung thromboembolism' OR 'microembolus, lung' OR 'pulmonary

embolism' OR 'pulmonary embolization' OR 'pulmonary embolus' OR 'pulmonary microembolism' OR 'pulmonary thromboembolic disease' OR 'pulmonary thromboembolism' OR 'thromboembolism, lung' OR 'deep vein thrombosis'/exp OR 'acute deep venous thrombosis' OR 'deep thrombophlebitis' OR 'deep vein thrombosis' OR 'deep venous thrombosis' OR 'deep venous thrombus' OR 'thrombosis, acute deep venous' OR 'venous thromboembolism'/exp OR 'thromboembolism, venous' OR 'vein thromboembolism'OR 'venous thromboembolism' OR 'blood clotting'/exp OR 'agglutination, blood' OR 'blood agglutination' OR 'blood clotting' OR 'blood clotting and blood clotting factors' OR 'blood clotting mechanism' OR 'blood clotting system' OR 'blood coagulation' OR 'blood coagulation system' OR 'clot formation' OR 'clotting contact phase' OR 'clotting system' OR 'clotting, blood' OR 'coagulation (blood)' OR 'coagulation system' OR 'coagulation,blood' OR 'hemocoagulation' OR 'thrombogenesis' OR 'thromb us formation'OR 'embolism'/exp OR 'embolism' OR 'embolism recurrence' OR 'embolus' OR embolic OR thrombolic) AND ('peripherally inserted central venous catheter'/exp OR 'lifecath picc expert' OR 'powerpicc solo catheter' OR 'spectrum turboject' OR 'peripherally inserted central catheter' OR 'peripherally inserted central venous catheter' OR 'pic line' OR 'picc line' OR 'indwelling catheter'/exp OR 'catheter a demeure' OR 'catheter indwelling' OR 'catheter, indwelling' OR 'catheter, indwelling, closed system' OR 'catheters, indwelling' OR 'closed system indwelling catheter' OR 'dauercatheter' OR 'indwelling cannula' OR 'indwelling catheter' OR 'indwelling catheter, closed system' OR 'self-retaining catheter')

ESM Text S3: Literature search strategy used for the web of science database

ALL=(Biomarkers OR biomarker OR biomarkers OR marker OR markers OR endpoint OR "end point" OR endpoints OR "end points" OR D-dimer OR haemoglobin OR hemoglobin OR "white cell" leukocyte OR leukocytosis OR "platelet count" OR "platelet counts" OR "clotting factor" OR "clotting factors" OR prothrombin OR P-selectin OR "blood count parameters" OR khorana OR neutrophil OR monocyte OR fibrin OR thrombopoietin OR "thrombin generation potential" OR fibrinogen OR "factor XIII" OR FXIII OR "factor VIII" OR FVIII OR c-reactive OR CRP OR "factor V Leiden" OR FVL OR RAM OR WBC OR cytokine OR cytokines OR chemokine OR chemokines OR "immune-related effectors" OR "acute-phase proteins" OR "reactive

oxygen" OR "reactive nitrogen" OR prostaglandin OR prostaglandins OR "cyclooxygenase-related factors" OR "transcription factor" OR "transcription factors" OR "growth factor" OR "growth factors" OR mGPS OR albumin OR NLR OR lymphocyte OR lymphocytes OR PLR OR SAA OR ROS OR RNS OR 3-Nitrotyrosine OR 8-oxodg OR 8-OHdG OR MDA OR HNE OR COX2 OR NF-κB] OR STAT OR "plasminogen activator inhibitor 1" OR PAI-1) AND ALL=(Thromboembolism OR Thrombosis OR "Pulmonary Embolism" OR thromboembolism OR thrombosis OR thrombus OR TE OR DVT OR VTE OR thrombii OR clot OR clots OR clotting or embolism OR thrombotic OR thrombolic OR embolic) AND ALL=(PICC OR PICCs OR “peripherally inserted central catheter” OR “peripherally inserted central catheters” OR “peripheral venous catheterization” OR “peripheral venous catheter” OR “peripheral venous catheters” OR "Catheterization, Peripheral".

ESM Figure S1: Funnel Plot for Platelets

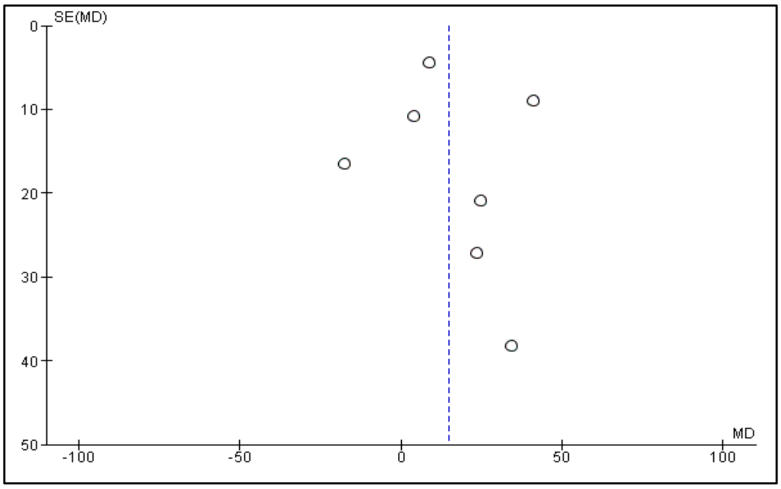

ESM Table S1: Characteristics of included studies in meta-analysis

**Study**	**Patient population**	**Age (years) - mean**	**Sample size DVT**	**Sample size non-DVT**	**Duration of PICC use (or time to thrombosis)**	**PICC indication**	**Trombosis history With DVT**	**Trombosis history without DVT**	**Cancer history with DVT**	**Cancer history without DVT**	**Biomarkers**
Ahn 2013	Patients with cancer (ambulatory and inpatient settings)	64±34.8	36	201	NR	Chemotherapy	NR	NR	Yes	Yes	Platelets, White blood cells.
Bhargava 2020	Medical-surgical intensive care unit	65±14.3	21	42	7.6 days	Multiple medications	3/21 (14.2%)	7/42 (16.7%)	2/21 (9.5%)	11/42 (26.1%)	Platelets.
Carmona 2021	Leukemia, children and adolescents	5 years (range 1–19 years)	26	49	NR	Chemotherapy	NR	NR	NR	NR	D-dimer, Platelets, White blood cells
Chen 2020	lung cancer	60.7 (+- 9.1)	55	693	NR	Chemotherapy	NR	NR	NR	NR	D-dimer, Fibrinogen, Platelets
Chopra 2017	Adult patients admitted to a general medicine ward or intensive care unit	(≥ 65 years - No DVT=49.2; Confirmed DVT= 49.3%) - (< 64 years - No DVT=50.8; Confirmed DVT= 50.7%)	475	22 535	NR	Chemotherapy or difficult intravenous access	3057 (13.6%)	113 (23.8%)	55 (11.6%)	1376 (6.1%)	International normalized ratio, Platelets, White blood cells

**Study**	**Patient population**	**Age (years) - mean**	**Sample size DVT**	**Sample size non-DVT**	**Duration of PICC use (or time to thrombosis)**	**PICC indication**	**Trombosis history With DVT**	**Trombosis history without DVT**	**Cancer history with DVT**	**Cancer history without DVT**	**Biomarkers**
Feng Zheng 2021	Cancer patients	57.43	82	386	NR	chemotherapy	37 (45.1%)	80 (20.7%)	17 (20.7%)	51 (13.2%)	Activated partial thromboplastin time, D-dimer, Platelet,

Study	Patient population	Age (years) - mean	Sample size DVT	Sample size non-DVT	Duration of PICC use (or time to thrombosis)	PICC indication	Trombosis history With DVT	Trombosis history without DVT	Cancer history with DVT	Cancer history without DVT	Biomarkers
											Prothrombin time, White blood cells
Hao 2017	Inpatient settings / oncology	55.8±13.9	86	2/3	85.9 (4-390)	Chemotherapy/ total parenteral nutrition	Yes (8.1%) No (91.9%)	Yes (2.6%) No (97.4%)	Yes	Yes	D-dimer, Fibrinogen, Fibrinogen Degradation Product, Platelets, White blood cells
Kang 2021	Inpatient, 88.7% had active solid cancer	51.1	56	2107	Median time to thrombosis = 14.5 days (range 1–302days).	Chemotherapy	NR	NR	43 (76.8)	1876 (89.0)	D-dimer
Li 2020	lymphoma patients	59+ - 8.52	55	165	NR	Chemotherapy	17%	1.8%	NR	NR	D-dimer, Platelets, White blood cells
Li 2021	Cancer Patients	52.5	165	2188	12 (range, 2–160) days - time to thrombosis	Chemotherapy	11 (6.7)	109 (5.0)	NR	NR	Activated partial thromboplastin time, Platelets, White blood cells
Liang 2018	Nonmetastatic nasopharyngeal carcinoma	With VTE 45.3±10.8<br>Without VTE 45.5± 11.5	217	2795	NR	Chemotherapy	NR	NR	Yes	Yes	Platelets, D-dimer, Fibrinogen, Fibrinogen Degradation Product
**Study**	**Patient population**	**Age (years) - mean**	**Sample size DVT**	**Sample size non-DVT**	**Duration of PICC use (or time to thrombosis)**	**PICC indication**	**Trombosis history With DVT**	**Trombosis history without DVT**	**Cancer history with DVT**	**Cancer history without DVT**	**Biomarkers**
Pan 2014	Inpatient settings	59.7± 14.1	11	148	1 week of insertion in 8 patients (72.73%); 2 weeks in 2 patients (18.18%); 4 months in 1 case (9.09%).	NR	NR	NR	NR	NR	Fibrinogen, Fibrinogen Degradation Product
Song 2020	Patients with cancer	Male 56.8±13.2<br>Female 52.9±14.8	59	280	NR	Chemotherapy	NR	NR	NR	NR	Platelets, D-dimer, White blood cells, Fibrinogen
Yu 2017	Lung cancer patients who underwent chemotherapy via PICC placement in hospital	Non PRVT 58.7±8.4 PRVT 60.2±9.7	23	324	13.56	Chemotherapy	No 16 (69.6%) Yes 7 (30.4%)	No 301 (92.9%) Yes 23 (7.1%)	Yes	Yes	Platelets, White blood cells

Yue Feng 2021	Lung cancer, inpatient	56.67	32	235	NR	Chemotherapy	NR	NR	5 (15.6%)	32 (52.6%)	Activated partial thromboplastin time, D-dimer, Fibrinogen, Fibrinogen Degradation Product, International normalized ratio, Platelets, Prothrombin time, White blood cells
**Study**	**Patient population**	**Age (years) - mean**	**Sample size DVT**	**Sample size non-DVT**	**Duration of PICC use (or time to thrombosis)**	**PICC indication**	**Trombosis history With DVT**	**Trombosis history without DVT**	**Cancer history with DVT**	**Cancer history without DVT**	**Biomarkers**
Zhang 2016	Patients with lymphoma and those with other types of cancer.	median age 52	249	7779	1 to 331 days, and the median of the interval was 25 days.	Continuous intravenous infusion or special irritant drugs, poor vascular elasticity or small blood vessel diameters	NR	NR	Yes	Yes	Platelets

DVT: deep vein thrombosis; NR: not reported; PICC: Peripherally Inserted Central Catheter; PRUEVT: PICC-related upper extremity venous thrombosis

ESM Table S2. Summary findings from included studies in qualitative analysis listed by biomarker

Biomarker	Author, year	Study design	Patient population	Sample size non-DVT	Sample size DVT	Outcome	Summary finding
**APTT**	Peng 2022	Retrospective cohort	breast cancer patients	1262	50	APTT (vs. per unit decrease)	**APTT was considered significant PICC-RVT predictive factor** OR: 7.112 (CI 1.278- 39.571) P= 0.025
**D-dímer**	Dubois, 2007	Prospective study	Children and adolescents (radiology department)	194	20	DVT risk (OR)	**No statistical significance (OR 1.73 CI95% 0.67–4.46; p= 0.25).** DVT vs. Non DVT > 2.0 ng/L (40% vs. 27.8%)
	Qian 2021	Prospective study	Patients with non-Hodgkin's lymphoma	317	53	Chi- square test	D-dimer >0.5 mg/L: Thrombosis group vs. Non-thrombosis 40 vs. 46 (p<0.001)
	Feng Zheng 2021	Prospective study	Cancer patients	386	82	Continuous	**No statistical significance (P=0.579)** D-dimer > 0.55 mg/L = PICC-RVT = 35 (42.7%); PICC-RVT=152 (39.4%);
	Liu 2021	Retrospective cohort	Clinical and surgical patients	3.093	769	Continuous	D-dimer negative (<0.5 mg/L) = 64% (95% CI: 58.2%–69.4%); D-dimer positive (≥0.5mg/L)= (36%, 95% CI: 30.6%-41.8%). Thirty-nine of 180 patients had VT despite having a negative D-dimer result, resulting in a failure rate of 21.7% (95% CI: 16.3%–28.3%). **The D-dimer levels maybe should not be used as a diagnostic index to rule out PICC-associated upper extremity vein thrombosis.**
**Biomarker**	**Author, year**	**Study design**	**Patient population**	**Sample size non-DVT**	**Sample size DVT**	**Outcome**	**Summary finding**
**D-dímer**	Peng 2022	Retrospective cohort	Breast cancer patients	1262	50	D-dimer (vs. per unit increase)	**D-dimer was considered significant PICC-RVT predictive factor** OR: 3.673 (CI 1.698 - 7.946) P= 0.001

**Fibrinogen**	Chen, 2015	Retrospective study	Patients with lung cancer at hospital	1500	38	DVT rate	**Statistical significance (OR: 2.055, CI95%: 1.070-3.764, p=0.028)**<br>Non DVT vs. DVT<br><2 g/L (3.39%)<br>2-4 g/L (1.67%)<br>>4 g/L (3.62%)
	Dubois, 2007	Prospective study	Children and adolescents (radiology department)	194	20	DVT risk (OR)	**No statistical significance OR 2.42 CI95% 0.78–7.53; p=0.12)**<br>DVT vs Non DVT<br>> 4.0 g/L (25% vs. 48.4%)
	Fu, 2019	Retrospective study	Lung cancer patients undergoing chemotherapy	160	40	Chi- square test	**Statistical significance (p=0.001)**<br>DVT vs. Non DVT<br>< 4 g/L (X^2 10.86)<br>≥ 4 g/L (X^2 10.86)
	Peng 2022	Retrospective cohort	breast cancer patients	1262	50	NR	FIB was not associated to PICC related to DVT
**Hemoglobin**	Chopra, 2017	Prospective study	Adult patients admitted to a general medicine ward or intensive care unit	475	22.535	Chi- square test	**Statistical significance (p<0.01)**<br>Non DVT vs. DVT<br>Non DVT 10.20 (8.80–11.70)<br>DVT 9.80 (8.60–11.50)
	Peng 2022	Retrospective cohort	Breast cancer patients	1262	50	NR	**HB was not associated to PICC related to DVT**
**Biomarker**	**Author, year**	**Study design**	**Patient population**	**Sample size non-DVT**	**Sample size DVT**	**Outcome**	**Summary finding**
**Hemoglobin**	Qian 2021	Prospective study	Patients with non-Hodgkin's lymphoma	317	53	Chi- square test	**Statistical significance (p<0.001)**<br>Hemoglobin <100 g/L: Thrombosis group vs. Non-thrombosis group 25 vs. 31
	Shi, 2014	Prospective study	Patients undergoing PICC chemotherapy	176	12	DVT rate	**No statistical significance (p ≥ 0.05).**<br>DVT vs. Non DVT <100 g/L
	Yuen 2021	Prospective study	cancer and non-cancer cohorts	246	18	Continuous	**No statistical significance (p=0.69)**<br>Cancer patient characteristics with VTE versus no VTE: Hemoglobin <100g/l, n (%) 1 (10) vs. 29 (22)
**HbA1c (% of Hg)**	Wilson, 2018	Case control study	Inpatient, whether the patient had a diagnosis of diabetes (yes/no)	584	116	Continuous	**No statistical significance (p = 0.84)**<br>DVT vs. Non DVT<br>DVT 7.3 (5.4-11.1, SD = 1.9)<br>Non DVT 7.6 (4.3-14.7, SD = 2.4)

**Platelet count**	Al-Asadi, 2019	Retrospective study/ cohort	Patients in the medical oncology unit	135	23	VTE risk (OR)	**No statistical significance (OR 1.01 CI95% 1.00- 1.01; p= 0.103)**.
	Chen, 2015	Retrospective study	Patients with lung cancer at hospital	1500	38	DVT rate	**No statistical significance (p= 0.459).** Non DVT vs. DVT <100× 10^9/L (2.44%) 100-300× 10^9/L (2.66%) >300× 10^9/L (1.13%)
	Dubois, 2007	Prospective study	Children and adolescents	194	20	DVT risk (OR)	**No statistical significance (OR 1.40 CI95% 0.49–3.99; p= 0.58)** DVT vs Non DVT > 440 × 10^9/L (55% vs 67.5%)
**Biomarker**	**Author, year**	**Study design**	**Patient population**	**Sample size non-DVT**	**Sample size DVT**	**Outcome**	**Summary finding**
**Platelet count**	Fu, 2019	Retrospective study	Lung cancer patients undergoing chemotherapy	160	40	Chi- square test	**No statistical significance (p=0.803).** DVT vs. Non DVT < 300×10^9/L (X^2 0.063) ≥ 300×10^9/L (X^2 0.063)
	Lin 2021	Retrospective cohort	Breast cancer	744	36	Chi- square test	**No statistical significance (P value =0.69)** Platelet number <200 ×109 with Venous Thrombosis = 20 (4.37%); Platelet number ≥200 ×109 with Venous Thrombosis= 16 (4.97%); (X^2 0.16);
	Peng 2022	Retrospective cohort	Breast cancer patients	1262	50	PLT (vs. per unit increase)	**PLT was considered significant PICC-RVT predictive factor** OR: 3.783 (CI: 1.756 - 8.149) P= 0.001
	Qian 2021	Prospective study	Patients with non-Hodgkin's lymphoma	317	53	Chi- square test	**No statistical significance (p= 0.315)** Platelets >350*109/L Thrombosis group vs. Non-thrombosis 6 vs. 23
	Shi, 2014	Prospective study	Patients undergoing PICC chemotherapy	176	12	DVT rate	**No statistical significance (p ≥ 0.05).** DVT vs. Non DVT >300 ×10^9/L

DVT: Deep vein thrombosis; PICC: Peripherally Inserted Central Catheter.

Biomarker	Author, year	Study design	Patient population	Sample size non-DVT	Sample size DVT	Outcome	Summary finding
	Yuen 2021	Retrospective single	Cancer and non-cancer cohorts	246	18	Continuous	**No statistical significance** (p=0.36) Cancer patient with VTE vs. no VTE: Platelet count <50x10^9/l on two occasions, n (%)vs. 22(17);
**Prothrombin fragment 1.2**	Carmona 2022	Prospective cohort study	Children with acute lymphoblastic leukemia	49	26	Continuous	**No statistical significance (p = 0.16).** VTE vs. Non VTE 377 (109, 1200) (pmol/L) vs. 316 (112, 1200)
**Biomarker**	**Author, year**	**Study design**	**Patient population**	**Sample size non-DVT**	**Sample size DVT**	**Outcome**	**Summary finding**
**Prothrombin time**	Chen, 2015	Retrospective study	Patients with lung cancer at hospital	1500	38	DVT rate	**No statistical significance (p=0.523).**<br>Non DVT vs. DVT<br><10 (n= 3.09%)<br>10-14 (n= 2.38%)<br>>14 (n= 6.67%)
	Fu, 2019	Retrospective study	Lung cancer patients undergoing chemotherapy	160	40	Chi- square test	**No statistical significance (p=0.722).**<br>DVT vs. Non DVT<br><10 g/L (X^2 0.126)<br>≥ 10 g/L (X^2 0.126)
**White blood cell**	Al-Asadi, 2019	Retrospective study/ cohort	Patients in the medical oncology unit	135	23	DVT risk (OR)	**No statistical significance (OR 0.86 CI95% 0.69- 1.08; p=0.202).**
	Jones, 2017	Retrospective study/ cohort	Ambulant cancer patients	463	27	DVT risk (OR)	**No statistical significance (OR 1.10 CI95% 0.95- 1.28, p=0.19)**
	Qian 2021	Prospective	Patients with non-Hodgkin's lymphoma	317	53	Chi- square test	**No statistical significance (p=0.324)**<br>White blood cell count >11*109/L Thrombosis group vs. Non-thrombosis 12 vs. 54
	Shi, 2014	Prospective study	Patients undergoing PICC chemotherapy in the Department of Surgical Oncology	176	12	DVT risk (OR)	**Statistical significance (OR 145.08 CI 95% 100.54-190.71; p < 0.001)**<br>DVT vs. Non DVT<br>>11.4 × 10^9/L
**Biomarker**	**Author, year**	**Study design**	**Patient population**	**Sample size non-DVT**	**Sample size DVT**	**Outcome**	**Summary finding**

	Yuen 2021	Retrospective	Cancer and non-cancer cohorts	246	18	Continuous	**No statistical significance (p=0.12)**<br>Cancer patient characteristics with VTE versus no VTE: Leukocyte count >12x109/l at PICC placement, n (%) 3 (30) vs. 16 (12)
**Thrombin–antithrombin complex**	Carmona 2022	Prospective cohort study	Children with acute lymphoblastic leukemia	49	26	Continuous	**No statistical significance (p = 0.32).**<br>VTE vs. Non VTE<br>6.4 (2.2, 60) µg/L vs. 5.6 (2.5, 27.1)

ESM Table S3: Quality of included studies using Newcastle Ottawa Scale for non-randomized studies

Study	**Retrospective (R) or prospective (P)**	**Selection**	**Comparability**	**Outcome**	**Score out of 9**	**Overall Quality**
Ahn 2013	R	★★★★	★★	★★★	9	High quality
Bhargava 2020	R	★★★★	★★	★★★	9	High quality
Carmona 2021	P	★★★★	★★	★★★	9	High quality
Chen 2020	R	★★★★	★★	★★★	9	High quality
Chopra 2017	P	★★★★	★★	★★★	9	High quality
Feng Zheng 2021	P	★★★★	★★	★★★	9	High quality
Hao 2017	R	★★★★	★★	★★★	9	High quality
Kang 2021	R	★★★★	★★	★★★	9	High quality
Li 2020	P	★★★★	★★	★★★	9	High quality
Li 2021	P	★★★★	★★	★★★	9	High quality
Liang 2018	R	★★★★	★★	★★★	9	High quality
Pan 2014	R	★★★★	★★	★★★	9	High quality
Song 2020	R	★★★★	★★	★★★	9	High quality
Yu 2017	P	★★★★	★★	★★★	9	High quality
Yue Feng 2021	P	★★★★	★★	★★★	9	High quality
Zhang 2016	R	★★★★	★★	★★★	9	High quality

ESM Table S4: Meta-regression with Activated partial thromboplastin time, D-dimer, fibrinogen and international normalized ratio, age and male gender

		R^2	P value	(95% CI)
D-dimer	age	7.57%	0.2387	(-0.0078-0.0313)
	gender male	0%	0.4342	(-0,0131-0.0304)
APTT	age	0%	0.6583	(0.3477-0.2197)
	gender male	0%	0.6660	(-0.0865-0.0553)
Fibrinogen	age	0%	0.4315	(-0.0303-0.0710)
	gender male	0%	0.2517	(-0.0071-0.0271)
INR	age	0%	0.9408	(-0.0130-0.0140)
	gender male	44.27%	0.1215	(-0.0004-00030)
